# Prevalence and genetic diversity of *Enterocytozoon bieneusi* in sheep in China

**DOI:** 10.1186/s13071-018-3178-9

**Published:** 2018-11-12

**Authors:** Heng Yang, Rongsheng Mi, Long Cheng, Yan Huang, Rui An, Yehua Zhang, Haiyan Jia, Xiaoli Zhang, Xu Wang, Xiangan Han, Zhaoguo Chen

**Affiliations:** 1 Key Laboratory of Animal Parasitology of Ministry of Agriculture, Laboratory of Quality and Safety Risk Assessment for Animal Products on Biohazards (Shanghai) of Ministry of Agriculture, Shanghai Veterinary Research Institute, Chinese Academy of Agricultural Sciences, Shanghai, 200241 China; 2 Animal Husbandry and Veterinary Bureau of Changji Hui Autonomous Prefecture, Xinjiang Uyghur Autonomous Region, Changji, 831100 Xinjiang China

**Keywords:** *Enterocytozoon bieneusi*, Sheep, Prevalence, Genotyping, China

## Abstract

**Background:**

*Enterocytozoon bieneusi* is a common species of microsporidia that not only influences human health but also threatens animal productive performance and value. However, there have been no systematic studies of the prevalence of *E. bieneusi* in sheep in China.

**Results:**

A total of 953 fecal specimens were collected from sheep from 11 provinces across five regions of China and analyzed for *E. bieneusi* by nested PCR targeting the ribosomal internal transcribed spacer (ITS). *Enterocytozoon bieneusi* infections were detected in four regions, with an overall infection rate of 20.4% (194/953). The highest infection rate was detected in pre-weaned lambs (25.0%), followed by post-weaned lambs (22.2%) and adult sheep (14.6%). *Enterocytozoon bieneusi* was found in nine of the 11 tested provinces, with infection rates between 2.9–51.7%. Eleven genotypes were identified based on ITS analysis, including seven known genotypes (BEB6, CHG1, CHG3, CHS7, CHS8, COS-I and NESH5) and four novel genotypes (CHHLJS1, CHHLJS2, CHNXS1 and CHXJS1). All 11 genotypes were clustered into group 2, and the zoonotic genotype BEB6 was the dominant genotype (*n* = 129, 66.5%) in sheep.

**Conclusion:**

The prevalence of *E. bieneusi* was studied in five regions representing most areas where sheep are bred in China. This is the first report of *E. bieneusi* infection in sheep for seven Chinese provinces. Geographical differences were detected in the distribution of *E. bieneusi* genotypes, but no differences were found among sheep in different age groups. The zoonotic genotype BEB6 was the dominant genotype, indicating that sheep are a potential source of zoonotic microsporidiosis in China. These results improve our knowledge of the epidemiology of *E. bieneusi* in sheep in China.

## Background

Microsporidia are obligate intracellular eukaryotic parasites with a wide range of hosts that includes arthropods, birds, mammals and humans [1, 2]. To date, more than 1300 microsporidian species belonging to 150 genera have been reported [3], including at least 14 microsporidian species belonging to eight families that have been reported to infect humans. The most common species, *Enterocytozoon bieneusi* [4, 5], can cause severe diarrhea in immunocompromised humans and animals, and zoonotic genotypes from domestic animals may be a threat to public health [1, 6, 7].

*Enterocytozoon bieneusi* has frequently been reported in domestic animals and wildlife all over the world [7]. Because it is difficult to distinguish *E. bieneusi* spores using microscopy, and no sophisticated culture approaches have been developed *in vitro*, molecular methods have been widely used for *E. bieneusi* detection [8]. To date, polymorphisms in the ribosomal internal transcribed spacer (ITS) of the rRNA gene have been widely used for *E. bieneusi* genotyping, and more than 200 *E. bieneusi* genotypes have been reported in humans and animals [8, 9]. When analyzed in combination with phylogeny, these genotypes can be grouped into several genetically isolated clusters [10]. Group 1 includes zoonotic genotypes that have been reported in humans and animals [11]. Groups 2 to 9 have mainly been reported in animals and wastewater [11, 12], and few genotypes have been detected in humans [8].

In addition to humans, *E. bieneusi* also infects a variety of animals, such as birds, cats, cattle, deer, dogs, donkeys, horses, pigs and wild mammals [5, 7, 13–20]. However, the epidemiology of *E. bieneusi* has rarely been reported in sheep. To date, a few studies have reported *E. bieneusi* infection in sheep in Brazil [21], China [19, 22–26], Iran [27] and Sweden [28] (Table 1). Several genotypes were identified in these studies, including genotypes D, BEB6, BEB7, EbpC, O, I, Peru6, NESH1-NESH 6, CS-4, CM7, COS-I-COS-VII and CHS1-CHS12 [7, 22, 23, 25]. Among these, genotypes BEB6, D, EbpC, O, I and Peru6 have been reported in humans [8, 21]. BEB6 is the most prevalent genotype detected in sheep in Brazil and China [21, 23–25].

**Table 1:** Prevalence of *E. bieneusi* and distribution of ITS genotypes in sheep based on published reports

Year	Country	Province (China)	Prevalence (%)	Genotype (no. of samples)	Reference
2014	Sweden		45.0 (49/109)	BEB6 (32), OEB1 (6), OEB2 (2), BEB6 + OEB1 (4), BEB6 + OEB2 (4), NDb (1)	[28]
2016	Brazil		19.2 (24/125)	BEB6 (11), BEB7 (8), I (2), BEB18 (1), BEB19 (1), LW1(1)	[21]
2014	China	Heilongjiang	4.4 (2/45)	BEB6 (2)	[19]
2015	China	Heilongjiang	13.9 (68/489)	BEB6 (28), CM7 (3), CS-4 (4), BEB6/CM7a (5), BEB6/OEB1a (5), BEB6/NESH4a (3), OEB1(3), BEB6/NESH6a (1), CS-4/EbpCa (1), NESH1(1), NESH2 (1), NESH3 (1), NESH5 (1),	[22]
2015	China	Heilongjiang	22.5 (31/138)	BEB6 (12), Peru6 (5), D (4), O (3), COS-I to COS-VII (one each),	[23]
2015	China	Inner Mongolia	69.3 (260/375)	BEB6 (237), CM7 (23)	[24]
2016	China	Henan	51.9 (161/310)	BEB6 (53), COS-I (12), CM4 (1), CHG3 (5), CHS3 (2), CHS4 (1), CHS5 (1), CHS6 (1), CHS10 (1), CHS12 (1)	[25]
		Liaoning	9.4 (6/64)	BEB6 (3)	
		Heilongjiang	25.0 (10/40)	BEB6 (4), COS-I (2), CHS7 (1), CHS8 (1), CHS9 (1), CHS11 (1)	
2018	China	Qinghai	23.4 (73/312)	BEB6 (31), COS-I (25), NESH5 (11), CHS17 (2), CHS13 (1), CHS14 (1), CHS15 (1), CHS16 (1)	[26]

In China, *E. bieneusi* infections have been reported in sheep in five provinces, including Heilongjiang, Henan, Inner Mongolia, Liaoning and Qinghai Province, with infection rates ranging between 4.4–69.3% [19, 22–26] (Table 1). At the end of 2016, China had the largest number of sheep in the world (http://www.fao.org/faostat/en/#data/QA), with most being bred in northern areas. However, there have been no systematic studies of *E. bieneusi* infection in sheep in China. Therefore, in order to determine the epidemiology and genetic diversity of *E. bieneusi* in sheep in China, 11 provinces across five regions that represent most areas where sheep are farmed in China were selected for *E. bieneusi* testing. These results lay a foundation for a better understanding of the epidemiology and genotypic features of *E. bieneusi* in China.

## Methods

### Fecal specimen collection

**Fig. 1:**
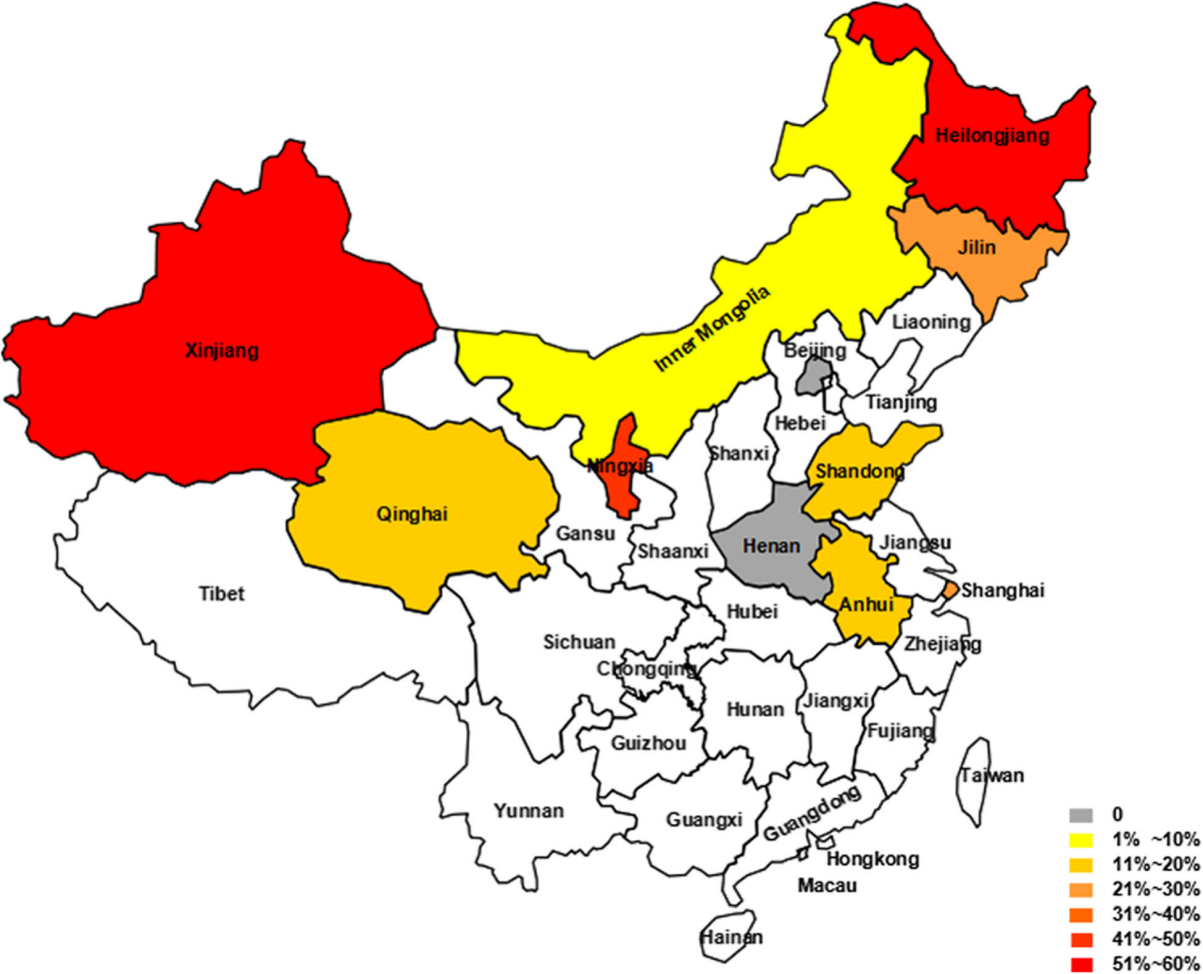
Geographical map of sampling provinces included in this study. The map was generated using Microsoft Office PowerPoint 2016 software

Between June 2013 and September 2015, a total of 953 fecal samples were collected from sheep in the following 11 provinces in China: Henan Province in central China; Anhui Province, Shandong Province and Shanghai City in eastern China; Beijing City and Inner Mongolia Autonomous Region in northern China, Heilongjiang Province and Jilin Province in northeast China; and Ningxia Hui Autonomous Region, Qinghai Province and Xinjiang Uyghur Autonomous Region in northwest China (Fig. 1). One fecal specimen was collected from each sheep. The three age groups selected for this study were pre-weaned (< 3 months) and post-weaned (3–12 months) lambs, and adult sheep (> 12 months). Fresh fecal specimens were collected from sheep using sterile gloves, and each specimen’s information was recorded, including location, date and age. Specimens were transported to the laboratory at low temperatures for further detection.

### Sample preparation and DNA extraction

Approximately 300 mg of each fecal specimen was transferred to a 50 ml sterile tube, 20 ml of sterile water was added, and the mixture then stirred with a 1 ml pipette tip to loosen the sample. A 200-300 μl sample was used for DNA extraction with a FastDNA SPIN Kit for Soil (MP Biomedicals, Santa Ana, CA, USA) according to the manufacturer’s instructions.

### PCR amplification

A 389 bp fragment of the *E. bieneusi* ITS gene was amplified by nested PCR as previously described by Buckholt et al. [29]. Briefly, a 435 bp PCR product was amplified using the primers EBITS3 (5'-GGT CAT AGG GAT GAA GAG-3') and EBITS4 (5'-TTC GAG TTC TTT CGC GCT C-3') for the primary PCR, and a 389 bp PCR product was amplified using the primers EBITS1 (5'-GCT CTG AAT ATC TAT GGC T-3') and EBITS2.4 (5'-ATC GCC GAC GGA TCC AAG TG-3') for the secondary PCR. Positive and negative controls were included in each PCR reaction. *Ex Taq* DNA Polymerase (TaKaRa Bio Inc., Beijing, China) was used for PCR amplifications. The secondary PCR products were examined by gel electrophoresis on a 1.2% agarose gel containing 4S Green Plus Nucleic Acid Stain (Sangon Biotech, Shanghai, China).

### Sequence analysis and phylogenetic construction

Positive samples from the second round of PCR gel electrophoresis were sequenced and analyzed using the BLAST program (http://blast.ncbi.nlm.nih.gov/Blast.cgi). Phylogenetic analysis of *E. bieneusi* was performed using MEGA 7.0 software [30]. All positive samples were sequenced twice, and novel genotypes were designated as previously described by Santín & Fayer [8]. Briefly, all sequences obtained in the present study were analyzed using the BLAST program to determine the *E. bieneusi* genotype based on 243 bp of the ITS gene region. Sequences that differed from published genotypes were considered new genotypes.

### Statistical analysis

Differences in infection rates between different regions, provinces and ages were assessed using a Chi-square test with SPSS Version 21.0 software (IBM Corp., Armonk, NY, USA). Differences were considered statistically significant when *P* < 0.05. Prevalence rates and 95% confidence intervals (CI) were also determined. Unique ITS sequences of *E. bieneusi* were submitted to the GenBank database under the accession numbers MH432644-MH432647.

## Results

### Overall prevalence and genotypic features of *E. bieneusi* in sheep

The overall prevalence of *E. bieneusi* infection in sheep in this study was 20.4% (194/953; 95% CI: 17.8–23.1%). All positive samples were sequenced for *E. bieneusi* genotype determination based on BLAST analyses. Eleven *E. bieneusi* genotypes were identified, including seven previously described genotypes (BEB6, CHG1, CHG3, CHS7, CHS8, COS-I and NESH5) and four novel genotypes (CHHLJS1, CHHLJS2, CHNXS1 and CHXJS1) (Table 2).

**Table 2:** Prevalence and genotype distribution of *E. bieneusi* in sheep from different regions of China

Region	No. of specimens	No. positive (%)	95% CI	Genotype (no. of samples)
Central China	35	0^d^	0–10.0	
Eastern China	326	58 (17.8)^c^	13.8–22.4	BEB6 (38), CHG1 (14), CHG3 (5), COS-I (1)
Northern China	166	3 (1.8)^d^	0.4–5.2	BEB6 (3)
Northeast China	130	50 (38.5)^a^	30.1–47.4	BEB6 (40), CHS7 (3), **CHHLJS1** (3), **CHHLJS2** (2), COS-I (2)
Northwest China	296	83 (28.0)^b^	23.0–33.5	BEB6 (48), CHS8 (32), **CHNXS1** (1), **CHXJS1** (1), NESH5 (1)
Total	953	194 (20.4)	17.8–23.1	BEB6 (129), CHS8 (32), CHG1 (14), CHG3 (5), CHS7 (3), COS-I (3), **CHHLJS1** (3), **CHHLJS2** (2), **CHNXS1** (1), **CHXJS1** (1), NESH5 (1)

*Note:* Different superscript letters within columns represent significant differences between groups (*P* < 0.05). Novel genotypes identified in this study are indicated in bold

Overall, BEB6 (*n* = 129) was the dominant genotype, followed by CHS8 (*n* = 32) and CHG1 (*n* = 14). All other genotypes were present in less than five specimens (Table 2).

### Prevalence of *E. bieneusi* in sheep in different regions of China

Of the five regions tested for *E. bieneusi* infections in sheep, northeast China had the highest infection rate (38.5%, 50/130; 95% CI: 30.1–47.4%), followed by northwest China (28.0%, 83/296; 95% CI: 23.0–33.5%), eastern China (17.8%, 58/326; 95% CI: 13.8–22.4%) and northern China (1.8%, 3/166; 95% CI: 0.4–5.2%). *Enterocytozoon bieneusi* infection was not detected in central China (Table 2). A significant difference was observed between different regions (*χ*^2^ = 82.562, *df* = 4, *P* < 0.0001). The most common genotype, BEB6, was detected in all *E. bieneusi*-positive regions. In northwest China, we found high prevalence rates of both BEB6 (57.8%, 48/83) and CHS8 (38.6%, 32/83) (Table 2).

### Prevalence of *E. bieneusi* in sheep in different provinces in China

Of the 11 provinces tested for *E. bieneusi* in sheep, the highest infection rate was detected in Heilongjiang (51.7%, 31/60; 95% CI: 38.4–64.8%), followed by Ningxia (47.1%, 57/121; 95% CI: 38.0–56.4%), Jilin (27.1%, 19/70; 95% CI: 17.2–39.1%), Shanghai (23.7%, 36/152; 95% CI: 17.2–31.3%), Xinjiang (19.2%, 19/99; 95% CI: 12.0–28.3%), Shandong (13.1%, 16/122; 95% CI: 7.7–20.4%), Anhui (11.5%, 6/52; 95% CI: 4.4–23.4%), Qinghai (9.2%, 7/76; 95% CI: 3.8–18.1%) and Inner Mongolia (2.9%, 3/102; 95% CI: 0.6–8.4%). *Enterocytozoon bieneusi* infection was not detected in Beijing (0/64; 95% CI: 0–5.6%) or Henan (0/35; 95% CI: 0–10.0%) (Table 3). Statistical analysis showed significant differences in infection rates of *E. bieneusi* among the different provinces (*χ*^2^ = 149.446, *df* = 10, *P* < 0.0001).

**Table 3:** Prevalence and genotype distribution of *E. bieneusi* in sheep from different provinces of China

Region	Province	No. of specimens	No. positive (%)	95% CI	Genotype (no. of samples)
Central China	Henan	35	0^e^	0–10.0	
Eastern China	Anhui	52	6 (11.5)^c^	4.4–23.4	CHG1 (3), CHG3 (3)
	Shandong	122	16 (13.1)^c^	7.7–20.4	BEB6 (3), CHG1 (11), CHG3 (2)
	Shanghai	152	36 (23.7)^b^	17.2–31.3	BEB6 (35), COS-I (1)
Northern China	Beijing	64	0^e^	0–5.6	
	Inner Mongolia	102	3 (2.9)^de^	0.6–8.4	BEB6 (3)
Northeast China	Heilongjiang	60	31 (51. 7)^a^	38.4–64.8	BEB6 (21), CHS7 (3), CHHLJS1 (3), COS-I (2), CHHLJS2 (2)
	Jilin	70	19 (27.1)^b^	17.2–39.1	BEB6 (19)
Northwest China	Ningxia	121	57 (47.1)^a^	38.0–56.4	BEB6 (24), CHS8 (32), CHNXS1 (1)
	Qinghai	76	7(9.2)^cd^	3.8–18.1	BEB6 (6), NESH5 (1)
	Xinjiang	99	19 (19.2)^bc^	12.0–28.3	BEB6 (18), CHXJS1 (1)
Total		953	194 (20.4)	17.8–23.1	

*Note:* Different superscript letters within columns represent significant differences between groups (*P* < 0.05)

The distribution of *E. bieneusi* genotypes differed among the different provinces. The most common genotype, BEB6, was detected in Heilongjiang (21/31), Inner Mongolia (3/3), Jilin (19/19), Qinghai (6/7), Shanghai (35/36) and Xinjiang (18/19). In contrast, CHG1 (11/16) and CHS8 (32/57) were the dominant genotypes in Shandong and Ningxia, respectively. Four novel genotypes were identified in Heilongjiang (*n* = 2), Ningxia (*n* = 1) and Xinjiang (*n* = 1). More than two genotypes were detected in most provinces, with the exception of Jilin and Inner Mongolia, where only one genotype (BEB6) was found (Table 3).

### Prevalence of *E. bieneusi* in different age groups of sheep

*Enterocytozoon bieneusi* infections were detected in all three age groups. The highest prevalence rate was detected in pre-weaned lambs (25.0%, 73/292; 95% CI: 20.1–30.4%), followed by post-weaned lambs (22.2%, 72/325; 95% CI: 17.8–27.1%) and adult sheep (14.6%, 49/336; 95% CI: 11.0–18.8%), with statistically significant differences among the age groups (*χ*^2^ = 11.438, *df* = 2, *P* = 0.003). More than six genotypes were identified in all age groups, and BEB6 was the most common genotype detected in each group (Table 4).

**Table 4:** Prevalence and ITS genotype distribution of *E. bieneusi* in different age groups of sheep

Age	No. of specimens	No. positive (%)	95% CI	Genotype (no. of samples)
Pre-weaned lambs	292	73 (25.0)^a^	20.1–30.4	BEB6 (54), CHS8 (12), CHG3 (2), COS-I (1), CHG1 (1), CHHLJS2 (1), CHNXS1 (1), CHXJS1 (1)
Post-weaned lambs	325	72 (22.2)^a^	17.8–27.1	BEB6 (47), CHS8 (11), CHS7 (2), COS-I (2), CHG1 (2), CHG3 (2), CHHLJS1 (2), CHSDS2 (2), NESH5 (1), CHHLJS2 (1)
Adult sheep	336	49 (14.6)^b^	11.0–18.8	BEB6 (28), CHS8 (9), CHG1 (9), CHS7 (1), CHG3 (1), CHHLJS1 (1)
Total	953	194 (20.4)	17.8–23.1	

*Note:* Different superscript letters within columns represent significant differences between groups (*P <* 0.05)

At the province level, infection rates of *E. bieneusi* differed by age group. The most susceptible age group was pre-weaned lambs, and *E. bieneusi* was detected in sheep in this age group in most provinces, including Inner Mongolia (8.1%, 3/31; 95% CI: 1.7–21.9%), Jilin (41.7%, 10/24; 95% CI: 22.1–63.4%), Ningxia (57.5%, 23/40; 95% CI: 40.9–73.0%), Qinghai (12.5%, 3/24; 95% CI: 2.7–32.4%), Shanghai (30.0%, 6/20; 95% CI: 11.9–54.3%) and Xinjiang (38.2%, 13/34; 95% CI: 22.2–56.4%). However, in two provinces, Anhui (13.6%, 3/22; 95% CI: 2.9–34.9%) and Heilongjiang (80.0%, 16/20; 95% CI: 56.3–94.3%), post-weaned lambs had the highest prevalence rate, and in Shandong Province (27.8%, 10/36; 95% CI: 14.2–45.2%) the group with the highest prevalence rate was adult sheep. With the exception of Anhui Province, BEB6 was detected in each group in all provinces (Table 5).

**Table 5:** Prevalence and ITS genotype distribution of *E. bieneusi* in different age groups from different provinces

Province	Age	No. of specimens	No. positive (%)	95% CI	Genotype (no. of samples)
Anhui	Pre-weaned lambs	16	2 (12.5)	1.6–38.4	CHG3 (2)
	Post-weaned lambs	22	3 (13.6)	2.9–34.9	CHG1 (2), CHG3 (1)
	Adult sheep	14	1 (7.1)	0.2–33.9	CHG1 (1)
Beijing	Pre-weaned lambs	20	0	0–16.8	
	Post-weaned lambs	22	0	0–15.4	
	Adult sheep	22	0	0–15.4	
Heilongjiang	Pre-weaned lambs	20	12 (60.0)	36.1–80.9	BEB6 (11), CHHLJS2 (1)
	Post-weaned lambs	20	16 (80.0)	56.3–94.3	BEB6 (9), CHS7 (2), COS-I (2), CHHLJS1 (2), CHHLJS2 (1)
	Adult sheep	20	3 (15.0)	3.2–37.9	BEB6 (1), CHS7 (1), CHHLJS1 (1)
Henan	Adult sheep	35	0	0–10.0	
Inner Mongolia	Pre-weaned lambs	37	3 (8.1)	1.7–21.9	BEB6 (3)
	Post-weaned lambs	35	0	0–10.0	
	Adult sheep	30	0	0–11.6	
Jilin	Pre-weaned lambs	24	10 (41.7)	22.1–63.4	BEB6 (10)
	Post-weaned lambs	25	6 (24.0)	9.4–45.1	BEB6 (6)
	Adult sheep	21	3 (14.3)	3.1–36.3	BEB6 (3)
Ningxia	Pre-weaned lambs	40	23 (57.5)	40.9–73.0	CHS8 (12), BEB6 (10), CHNXS1 (1)
	Post-weaned lambs	40	17 (42.5)	27.0–59.1	CHS8 (11), BEB6 (6)
	Adult sheep	41	17 (41.5)	26.3–57.9	CHS8 (9), BEB6 (8)
Qinghai	Pre-weaned lambs	24	3 (12.5)	2.7–32.4	BEB6 (3)
	Post-weaned lambs	32	3 (9.4)	2.0–25.0	BEB6 (2), NESH5 (1)
	Adult sheep	20	1 (5.0)	0.1–24.9	BEB6 (1)
Shandong	Pre-weaned lambs	57	1 (1.8)	0–9.4	CHG1 (1)
	Post-weaned lambs	29	5 (17.2)	5.9–35.8	BEB6 (2), CHG1 (2), CHG3 (1)
	Adult sheep	36	10 (27.8)	14.2–45.2	CHG1 (8), BEB6 (1), CHG3 (1)
Shanghai	Pre-weaned lambs	20	6 (30.0)	11.9–54.3	BEB6 (5), COS-I (1)
	Post-weaned lambs	64	17 (26.6)	16.3–39.1	BEB6 (17)
	Adult sheep	68	13 (19.1)	10.6–30.5	BEB6 (13)
Xinjiang	Pre-weaned lambs	34	13 (38.2)	22.2–56.4	BEB6 (12), CHXJS1 (1)
	Post-weaned lambs	36	5 (13.9)	4.7–29.5	BEB6 (5)
	Adult sheep	29	1 (3.5)	0.1–17.8	BEB6 (1)

### Phylogenetic analysis

**Fig. 2:**
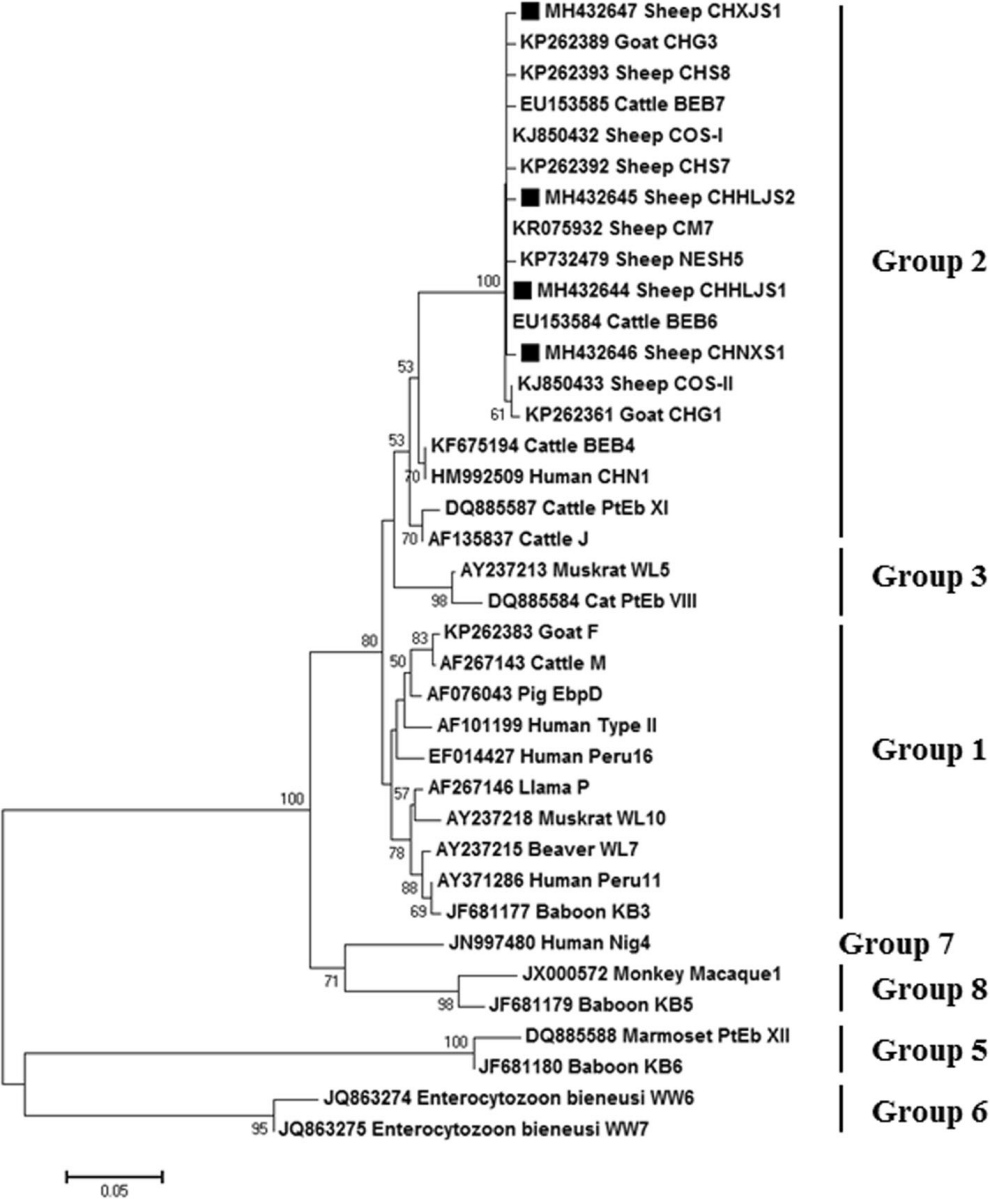
Phylogenetic analysis of *E. bieneusi* based on ITS gene sequences. The evolutionary history was inferred using the neighbor-joining method and evolutionary analyses were conducted in MEGA 7.0. Bootstrap values > 50% are shown. Filled squares indicate the novel genotypes obtained in the present study

Phylogenetic trees were constructed using the novel and known genotypes based on ITS nucleotide sequences as shown in Fig. 2. The results indicated that all genotypes detected in this study belonged to group 2.

## Discussion

In the present study, an investigation of *E. bieneusi* was conducted in 11 provinces, which included most areas where sheep are farmed in China. *Enterocytozoon bieneusi* infection was detected in nine provinces, suggesting that *E. bieneusi* is widespread in sheep in China. Compared to studies that used the same testing method, the overall prevalence of *E. bieneusi* in sheep in this study was 20.4%, which is similar to previous reports of *E. bieneusi* in sheep in Brazil (19.2%) [21], but lower than prevalence rates reported in Sweden (68.1%) [28]. The prevalence rate in this study was also higher than a previous report from Iran (10.0%), which was based on the *SSU* rRNA gene [27] (Table 1). Additionally, the prevalence rate of *E. bieneusi* in Heilongjiang in the present study (51.7%) was higher than the previously reported rate of 22.5% found using the same method [23]. However, compared to studies that used different ITS primers, prevalence rates in the present study in Henan (0%), Inner Mongolia (2.9%) and Qinghai (9.2%) were lower than previous reports in Henan (51.9%) [25], Inner Mongolia (69.3%) [24] and Qinghai (23.4%) [26], but higher than previous reports in Heilongjiang (4.4, 13.9 and 25.0%) [19, 22, 25]. The differences between our study and previous studies may be due to differences in detection methods, sampling sites, sampling seasons, sheep age groups or sheep densities. Prevalence rates of *E. bieneusi* in the other provinces, including Ningxia (47.1%), Jilin (27.1%), Shanghai (23.7%), Shandong (13.1%), Xinjiang (19.2%), Anhui (11.5%) and Beijing (0%), have not been previously reported.

In the present study, the prevalence of *E. bieneusi* was assessed in five regions, which include most areas where sheep are bred in China. We did not obtain samples from southeast or southwest China, mainly because goats are the dominant livestock in these areas. The prevalence of *E. bieneusi* in northeast China (38.5%) was higher than previously reported (13.9%) [22]. This may be due to differences in sampling sites or sheep age groups. In the present study, we did not detect *E. bieneusi* infection in central China, possibly because only adult sheep were tested in this area (Table 5). Similar results were also reported in China and Sweden [19, 28], where *E. bieneusi* infections were not detected in adult sheep. Therefore, future studies need to increase the number of specimens collected in this area.

In the present study, a higher prevalence of *E. bieneusi* was detected in lambs (23.5%) than in adults (14.6%), which is consistent with many previous studies in Brazil and China [21, 22, 24–26]. The prevalence of *E. bieneusi* in lambs under one year of age was 23.5%, which is similar to the findings of a previous study in Heilongjiang (18.2%) [22], but lower than reported in previous studies in other parts of China [25, 26] and in Sweden [28]. The prevalence in pre-weaned lambs (25.0%) was also similar to a study by Li et al. [19] in Heilongjiang (20.0%) but was significantly lower than previous reports by Ye et al. [24] in Inner Mongolia (77.8%). We also observed differences in adult sheep between this study and previous studies; the reported prevalence in this study (14.6%) was higher than that reported in Brazil (11.1%) by Fiuza et al. [21] and in China (8.7%) by Jiang et al. [22], but was lower than the prevalence reported in Inner Mongolia (62.9%) [24] and in Qinghai (22.7%) [26]. Another report also found a higher prevalence of *E. bieneusi* (39.4%) in adult sheep in Liaoning, Henan and Heilongjiang [25]. In contrast, two studies reported no *E. bieneusi* infection in adult sheep in Sweden [28] or Heilongjiang [19]. From these results, it seems that sheep age can affect the *E. bieneusi* infection rate in different regions.

The first reported *E. bieneusi* genotype, BEB6, was described in cattle in the USA [14]. Since then, this genotype has become common in ruminants, including sheep [19, 21–26, 28], goats [31], golden takins [32], deer [20], sika deer [20] and alpacas [33]. In addition, this genotype has also been found in humans [34], cats [15], rhesus macaques [16], yaks [26], horses [35], ducks and geese [36], as well as in urban wastewater [37, 38]. In this study, BEB6 was the most prevalent genotype (66.5%) and was detected in all provinces where *E. bieneusi* infection was found. These results are consistent with previous reports in the same provinces of China, including Heilongjiang [19, 22, 23, 25], Inner Mongolia [24] and Qinghai [26]. Other studies have also reported that BEB6 is the most common genotype in sheep in Sweden [28] and Brazil [21]. In China, BEB6 was also reported to be the most prevalent genotype in other provinces, such as Liaoning and Henan [25]. According to these results, we suggest that BEB6 is a dominant genotype with a widespread geographical distribution in sheep.

In this study, we identified five genotypes (CHG3, CHS7, CHS8, COS-I and NESH5) that were previously reported in sheep in China. CHG3 was previously detected in Henan [25]; CHS7 and CHS8 were previously detected in Heilongjiang [25]; COS-I was previously reported in Heilongjiang [23, 25], Henan [25] and Qinghai [26]; and NESH5 was previously found in Heilongjiang [22] and Qinghai [26]. Similar to these studies, we also found CHS7 and COS-I in Heilongjiang and NESH5 in Qinghai. However, we did not detect genotypes that had been previously reported in some provinces, such as genotype CM7, which was reported in Inner Mongolia and Heilongjiang [22, 24]. Although genotype CHG1 was previously reported in goats in Chongqing, Henan, Shaanxi and Yunnan [25], this study is the first report of this genotype in sheep. Phylogenetic analysis showed that all genotypes belonged to group 2. Although it has been classified as a cattle-specific group [11], some group 2 genotypes have been found in humans, including genotypes BEB6, I and J.

Based on known *E. bieneusi* genotypes [8], four novel genotypes were identified in sheep in this study. Compared to the known genotype BEB6, the novel genotypes CHHLJS1, CHNXS1 and CHXJS1 had two substitutions (A to T at position 155 and A to T at position 178), one substitution (A to G at position 135) and one substitution (A to G at positions 181), respectively. Compared to genotype CHS3, CHHLJS1 had one substitution (G to A at position 86). Phylogenetic analysis showed that all novel genotypes belonged to group 2.

According to the results of our study, there were differences in the geographical distribution of ITS genotypes in sheep. Although BEB6 was the most common genotype in most provinces, we found that CHS8 (56.1%) was the dominant genotype in Ningxia and CHG1 (68.7%) was the dominant genotype in Shandong. In addition, equal occurrences of CHG1 (*n* = 3) and CHG3 (*n* = 3) were found in Anhui. In most provinces, more than two genotypes were detected. Similar to previous studies, which reported several genotypes in Heilongjiang [22, 23, 25], we found more than five genotypes in this province (Table 1). Differences in the geographical distribution of *E. bieneusi* genotypes have also been reported in other studies. For example, BEB6 was the only genotype reported in Heilongjiang and Liaoning [19, 25], and only two genotypes (BEB6 and CM7) were previously reported in Inner Mongolia [23]. Moreover, more than five genotypes have been reported in Brazil [21], China [22, 23, 25, 26] and Sweden [28]. However, it seems that the distribution of ITS genotypes was not related to sheep age. In this study, more than six genotypes were detected in all age groups, and the distribution of ITS genotypes was similar between the groups, which is consistent with a previous study in Inner Mongolia [23].

*Enterocytozoon bieneusi* is an important protozoan parasite that is transmitted *via* water and food. Humans can become infected with microsporidia through human or animal fecal contamination in soil or water [39, 40]. In China, the *E. bieneusi* genotype BEB6 has been detected in wastewater from five cities [37, 41]. In the present study, BEB6 was the most prevalent genotype, indicating that sheep may be a source of *E. bieneusi* contamination in wastewater. However, whether *E. bieneusi* is present in wastewater near farms in these areas is unclear, and more studies are required to further understand the transmission of *E. bieneusi* between sheep and water.

## Conclusions

In this study, we assessed the prevalence and genetic diversity of *E. bieneusi* in sheep from 11 provinces across five regions of China. *E. bieneusi* was found in nine provinces, suggesting that *E. bieneusi* is widespread in sheep in China. The overall infection rate was 20.4%, and the highest infection rate was detected in pre-weaned lambs. At the province level, the prevalence in different age groups also differed. Eleven genotypes were detected in sheep in this study, including four novel genotypes. The zoonotic genotype BEB6 was the dominant genotype and may pose a potential threat to humans. We also observed geographical differences in the genotypic features of *E. bieneusi* in sheep, but no differences were found in genotypes among the different age groups. This study covered most areas of China where sheep are bred, and for seven of the provinces this is the first report of *E. bieneusi*. Therefore, this study increases our understanding of the prevalence and genotypic characterization of *E. bieneusi* in sheep in China.

## Abbreviations

ITS: Internal transcribed spacer
PCR: Polymerase chain reaction
*SSU* rRNA: Small subunit ribosomal RNA
